# Verification of a novel stress path method by true-triaxial test

**DOI:** 10.1038/s41598-024-56435-1

**Published:** 2024-03-13

**Authors:** Zhigang Ma, Xuefeng Li, Longlong Lv

**Affiliations:** 1 School of Civil and Hydraulic Engineering, Ningxia University, Yinchuan, 750021 China; 2 Solid Mechanics Institute, Ningxia University, Yinchuan, 750021 China

**Keywords:** Aeolian sand, Anisotropy, Triaxial test, Intermediate principal stress, Deviatoric plane, Solid Earth sciences, Engineering, Civil engineering

## Abstract

To verify the novel method of achieving a true-triaxial stress path with the pseudo-triaxial apparatus, a series of drained and undrained tests were carried out for the identical scheme with pseudo-triaxial apparatus and true-triaxial apparatus respectively. The differences between the two types of tests were quantified. The results show that the novel method effectively achieved the true-triaxial stress path by controlling the loading ratio of the pseudo-triaxial apparatus. The relationships of *q* − *ε*_1_ and *η* − *ε*_s_ measured by the two apparatuses had a higher similarity which decreases slightly with the *b* increase. When 0 ≤ *b* < 0.5, the slope of the critical state line measured by both apparatuses was almost identical. When 0.5 ≤ *b* ≤ 1, the slope of the critical state line measured by the novel method was slightly lower, but the biggest change was within 10% compared with the two Mohr–Coulomb criteria, the peak strength measured by the two apparatuses was distributed near the criteria, indicating the feasibility and rationality of the novel method. The tests show that the novel method greatly enriches the test range of pseudo-triaxial apparatus, which not only simplifies the process of soil 3D testing but also reduces the test cost.

## Introduction

The soil, as a fragmented granular material, shows remarkable anisotropy. Because the shape, arrangement and contact pattern of soil particles are markedly different in micro, the mechanical properties of each direction are evidently diverse. So anisotropy is of great significance to the development of microscopic mechanisms and its engineering applications. Although the true-triaxial apparatus is the most ideal device to reveal the mechanism of orthotropic deformation and strength for soils^1^, its shortcomings such as the collision of rigid loading have an unforeseeable impact on its application and the accumulation of test results.

True triaxial test plays an irreplaceable role in determining soil anisotropy. Anisotropy is affected by stress state, stress history and stress path, especially by intermediate principal stress *σ*_2_ or intermediate principal stress coefficient* b*, which increases the difficulty of exploring its mechanisms. To characterize the effect of *σ*_2_ on the soil mechanical behaviour, many scholars have conducted numerous true-triaxial tests^2,3^. Rodriguez^4^ studied the effects of cross-anisotropy on fine Nevada sand. Nakai^5^, Reddy^6^, Matsuoka^7^ and Choi^8^ designed tests with generalized normal stress as a constant for different types of soil, and the results show that the deformation is directly bound up with the stress path in three-dimensional space. Zhang^9^ and Xu^10^ have studied lateral deformation under different stress states in the shear process, which shows that lateral deformation is distinct under specific stress paths. Oda^11^ and Yamada^12^ studied the influence of structure on anisotropy degree by preparing horizontal sediment sand specimens. Ibsen^13^, Lade^14^ and Sayao^15^ studied the effect of *b* on the stress–strain relationships, and the results show remarkable anisotropy. The tests by Alshibli and Williams^16^, Chen^17^ and Li^18,19^ also state that the stress–strain relationships are dissimilar, and the anisotropy is affected. In particular, Verdugo^20^ and Riemer Seed^21^ found that the critical state of sand is not unique due to the influence of *b* and anisotropy, which is contrary to the critical state theory. In addition, Yao^22^ established a three-dimensional anisotropic sand UH model considering the effects of anisotropy and σ_2_. Li^23^ proposed the anisotropic critical state theory considering the effect of anisotropic fabric. Jiang^24^ established the constitutive theory of structural sand considering anisotropy based on the microscopic damage mechanism. The verification of these experimental views and the verification of the constitutive theory needs the true-triaxial test results as evidence. Although the true-triaxial test results have been applied to the academic and engineering fields, the software and hardware of the apparatus need to be improved gradually, which increases the difficulty of accurately determining anisotropic parameters^25–27^.

In response to the above questions, Li and Ma^28^ presented a method to achieve the true-triaxial stress path equivalent by pseudo-triaxial apparatus (PTA) and conducted a series of triaxial tests^29^. Although the proposed method is not a true-triaxial test in the real sense and it is an equivalent method to make up for the difficulties of true-triaxial application from the perspective of software, it will expand the test range of PTA and greatly reduce costs if the method can be popularized. It is regretful that the detailed description of the method is beyond the scope of this article, but if readers are interested in this method, please refer to the paten^28^. The main task here is quantifying the difference between the presented method and the true-triaxial test and verifying its rationality and practicability. Therefore, in this paper, the pseudo-triaxial test and true-triaxial tests on aeolian sand were conducted at an identical stress path respectively to quantify the difference between the two type tests and verify the rationality and validity of the novel method of achieving a true-triaxial stress path with the pseudo-triaxial apparatus, so as to make a reasonable correction.

## Test process

### Introduction to the presented method

To account for the advantages and disadvantages of cylindrical and rectangular specimens, the authors presented a method to achieve a true-triaxial stress path using PTA^28^. By controlling average principal stress *p*, generalized shear stress *q* and stress Lode angle *θ*_σ_, true triaxial apparatus (TTA) can achieve arbitrary stress paths in 3D space. Although the PTA cannot directly achieve the above stress path, the true-triaxial stress path in the generalized stress space (*p-q* space) can be consistent with the pseudo-triaxial stress path equivalently. Therefore, based on this feature, the arbitrary stress path as the TTA was achieved by the PTA equivalently.

The following describes one of the loading paths and adopts PTA and TTA for test verification respectively. The loading method is to keep the pseudo-triaxial stress path consistent with the true-triaxial stress path with *p* as a constant in the *p* − *q* space, so as to achieve the loading of the constant *p* stress path under different *b*. The specific control equation is derived as follows.

The average principal stress increment d*p*, generalized shear stress increment d*q* and *b* under true-triaxial condition are as follows.

1 $$\left\{ \begin{gathered} {\text{d}}p = \frac{{{\text{d}}\sigma_{1} + {\text{d}}\sigma_{2} + {\text{d}}\sigma_{3} }}{3} \hfill \\ {\text{d}}q = \frac{{\left( {2{\mkern 1mu} \sigma_{1} - {\mkern 1mu} \sigma_{2} - \sigma_{3} } \right){\text{d}}\sigma_{1} - \left( {{\mkern 1mu} \sigma_{1} - 2{\mkern 1mu} \sigma_{2} + {\mkern 1mu} \sigma_{3} } \right){\text{d}}\sigma_{2} - {\mkern 1mu} \left( {\sigma_{1} + \sigma_{2} - 2{\mkern 1mu} \sigma_{3} {\mkern 1mu} } \right){\text{d}}\sigma_{3} }}{{\sqrt 2 \sqrt {\left( {\sigma_{1} - \sigma_{2} } \right)^{2} + \left( {\sigma_{2} - \sigma_{3} } \right)^{2} + \left( {\sigma_{1} - \sigma_{3} } \right)^{2} } }} \hfill \\ b = \frac{{{\text{d}}\sigma_{2} - {\text{d}}\sigma_{3} }}{{{\text{d}}\sigma_{1} - {\text{d}}\sigma_{3} }} \hfill \\ \end{gathered} \right.$$

where *σ*_1_ is the major principal stress; *σ*_2_ is the intermediate principal stress; *σ*_3_ is minor principal stress; d*σ*_1_ is the major principal stress increment; d*σ*_2_ is the intermediate principal stress increment; d*σ*_3_ is minor principal stress increment.

It can be obtained from Eq. (1).

2 $${\text{d}}p = \frac{{\left( {1 + b} \right){\text{d}}\sigma_{1} + \left( {2 - b} \right){\text{d}}\sigma_{3} }}{3}$$

According to Eq. (2), when the mechanical response on the deviatoric plane needs to be measured for TTA, it is ensured that *p* and *b* are constant values during the shear process, i.e. d*p* = 0.

3 $${\text{d}}\sigma_{3} = \frac{b + 1}{{b - 2}}{\text{d}}\sigma_{1}$$

Equation (3)is the stress path control equation of true-triaxial under different *b*. However, if *b* is regarded as a proportional coefficient, the loading of *σ*_1_ and *σ*_3_ is controlled by PTA. As can be drawn from Eq. (3), if *σ*_1_ is controlled by PTA, the proportional increase and decrease of *σ*_3_ can be equivalent to achieving a constant* p* stress path consistent with TTA in *p* − *q* space.

## Apparatus introduction

**Figure 1:**
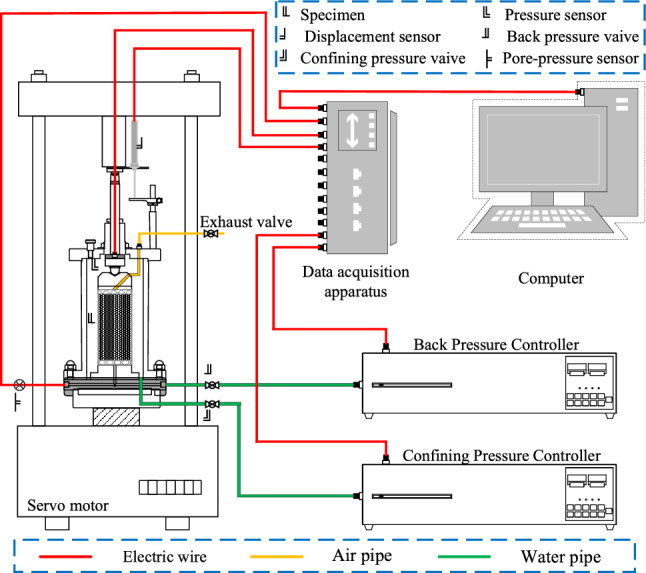
SLB-1 pseudo-triaxial apparatus.

**Figure 2:**
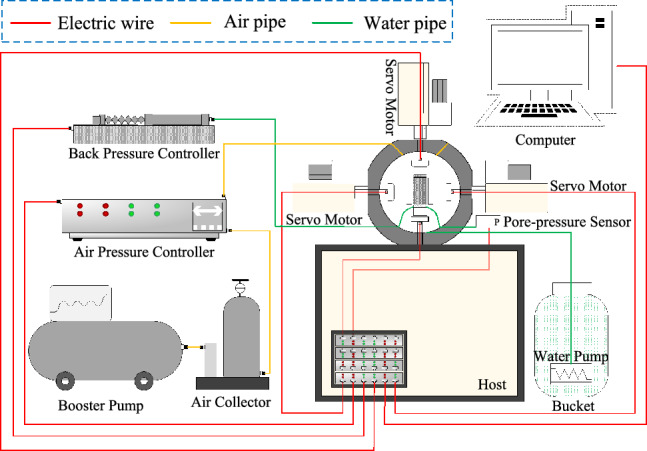
GDS-TTA true-triaxial apparatus.

The apparatus is SLB-1 pseudo-triaxial apparatus and GDS-EMTTA true-triaxial apparatus. The apparatus diagram is shown in Figs. 1 and 2. The PTA is a multifunctional flexible triaxial apparatus, which adopts servo motor rigid loading in the axial direction and hydraulic flexible loading in the radial direction. The apparatus can be loaded by stress or strain control, which can meet the needs of most static tests and can almost perfectly achieve various plane stress path tests.

**Figure 3:**
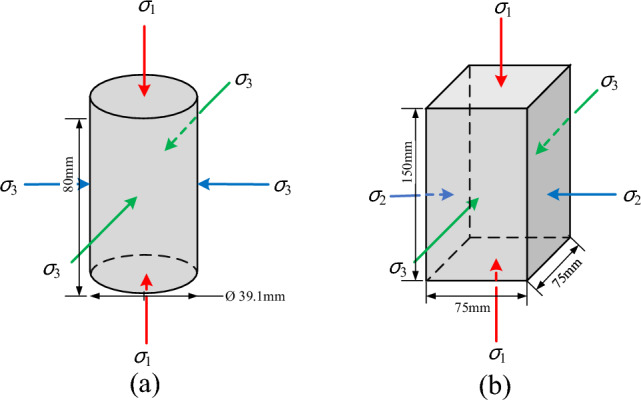
Stress analysis diagram of pseudo-triaxial and true-triaxial specimens: (**a**) Pseudo-triaxial specimen; (**b**) True-triaxial specimen.

The TTA is a rigid-flexible composite apparatus. The rigid loading is adopted in the *σ*_1_ and *σ*_2_ direction, and the pneumatic flexible loading is adopted in the *σ*_3_ direction, which can achieve multiple dynamic and static plane and spatial stress path tests. Moreover, the PTA adopts a cylindrical specimen with a size of *Φ*39.1 mm × 80 mm. The TTA adopts a rectangular specimen with a size of 75 mm × 75 mm × 150 mm. The stress state of a cylinder and rectangular specimen is shown in Fig. 3.

### Test materials and specimen preparation methods

**Figure 4:**
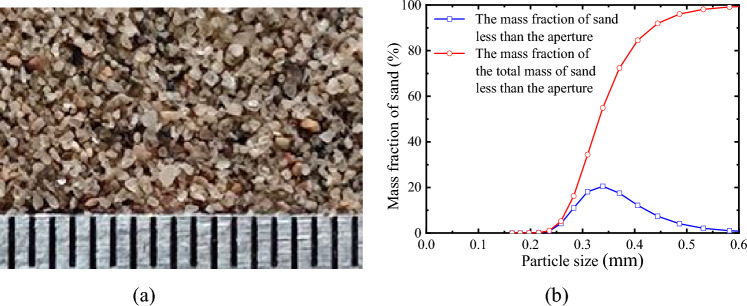
Tested material: (**a**) Mesoscopic image of the aeolian sand (0.5 mm/grid); (**b**) Particle size distribution ^29^_._

The material is the aeolian sand of Tengger Desert, and its mesoscopic image and particle size distribution are shown in Fig. 4a,b, respectively. According to the sieving method, the particle size range is 0.075 ~ 0.5 mm, the coefficient of curvature is 0.97, and the coefficient of nonuniformity is 1.42. According to the Standard for Engineering Classification of Soil (GB/T 50145-2007), the sand is classified as fine sand and poorly graded sand. For other physical parameters, please refer to reference 18.

Multiple sieving pluviation is adopted for specimen preparation. The cylindrical specimen is prepared in 5 layers and the rectangular specimen is prepared in 10 layers. We control the relative density to 0.37 and prepare the specimen with a dry density of 1.49 t/m^3^, corresponding to a porosity of 0.79. However, due to the small and uniform particle size, the multiple sieving pluviation under gravity is not conducive to the void formation, so it is difficult to make loose specimens for dry sand. The natural moisture content of aeolian sand is 0.14%. After attempts, it has been shown that the preparation of loose sand specimens can be effectively completed when the moisture content of aeolian sand is 0.7%. Therefore, the dry sand was subjected to moisture treatment. An appropriate amount of water is added to artificially mix the moisture content to 0.7%, stirred evenly, and stored in a sealed box for later use. The aeolian sand is used for specimen preparation only after moisture treatment. After the above progress, 20 kPa negative pressure is applied by a vacuum pump to the inside of the specimen through the vent hole of the specimen cap to maintain its shape and size. After the installation, pre-apply the confining pressure of 20 kPa and remove the negative pressure to complete the specimen preparation process. Then, saturation and consolidation tests are carried out. Both saturation and consolidation will have an important influence on the test results, Therefore, it is necessary to keep the parameters of each group test consistent before carrying out the triaxial test.

### Test scheme

**Figure 5:**
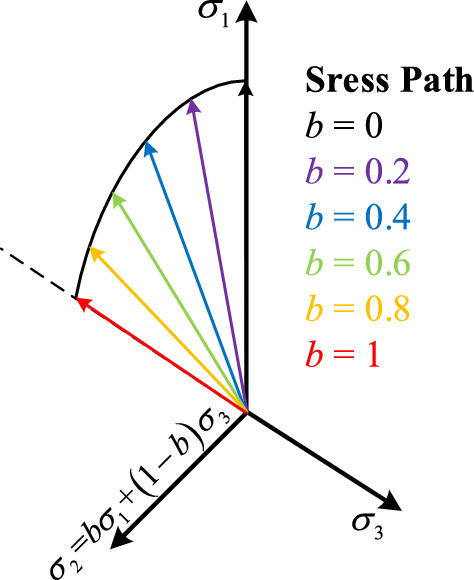
Schematic diagram of stress path in principal stress space.

To distinguish the difference between the test results loaded by PTA and the test results loaded by TTA, triaxial drained (CD) and undrained (CU) tests with *p* as constant (100, 300 and 600 kPa respectively) and different *b* (0, 0.2, 0.4, 0.6, 0.8, 1 respectively) are designed. The stress path in the principal stress space is shown in Fig. 5. The crucial parameters of the scheme are shown in Table 1.

**Table 1:** Crucial parameters of triaxial test with constant *p.*

Test material	Effective average principal stress *p* (kPa)	Intermediate principal stress coefficient *b*		Apparatus	Load method	Drained conditions	End condition
Aeolian sand	100	0	0.2	I Pseudo-triaxial apparatus II True-triaxial apparatus	Stress control 1 kPa/min	I Drained II Undrained	Strain 15%
	300	0.4	0.6				
	600	0.8	1				

The PTA controls the loading by setting the ratio of *σ*_1_ and *σ*_3_ according to Eq. (3), and the scheme in Table 1 can be achieved by calculating according to the formula *σ*_2_ = *bσ*_1_ + (1 − *b*)*σ*_3_. The TTA can control the target values of the stress states of *σ*_1_, *σ*_2_ and *σ*_3_, thus achieving the test scheme in which *p* is a constant. The target value of the subordinate stress state is calculated as follows.

4 $$p = \frac{{\sigma_{1} + \sigma_{2} + \sigma_{3} }}{3}$$

5 $$b = \frac{{\sigma_{2} - \sigma_{3} }}{{\sigma_{1} - \sigma_{3} }}$$

It can be obtained from Eqs. (4) and (5).

6 $$\left\{ \begin{gathered} \sigma_{1} = \frac{{3p - \left( {2 - b} \right)\sigma_{3} }}{1 + b} \hfill \\ \sigma_{2} = \frac{{3bp + \left( {1 - 2b} \right)\sigma_{3} }}{1 + b} \hfill \\ \end{gathered} \right.$$

**Figure 6:**
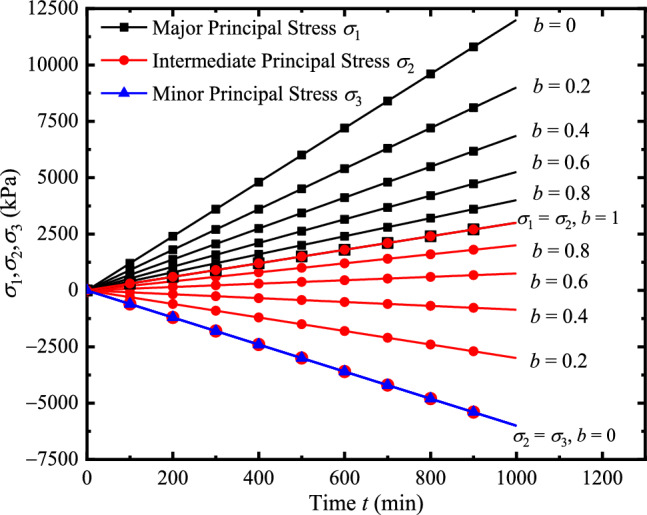
The loading path diagram under different *b.*

Given *p* and *b*, given the decrement of *σ*_3_ in unit time, we can obtain the decrement of *σ*_1_ and the increment of *σ*_2_. Similarly, the target values of the other two principal stresses can also be calculated by assuming the change of *σ*_1_ or *σ*_2_ in unit time. The loading path diagram under different *b* is shown in Fig. 6.

## Comparative analysis of true-triaxial test and pseudo-triaxial test

### Stress path comparison

**Figure 7:**
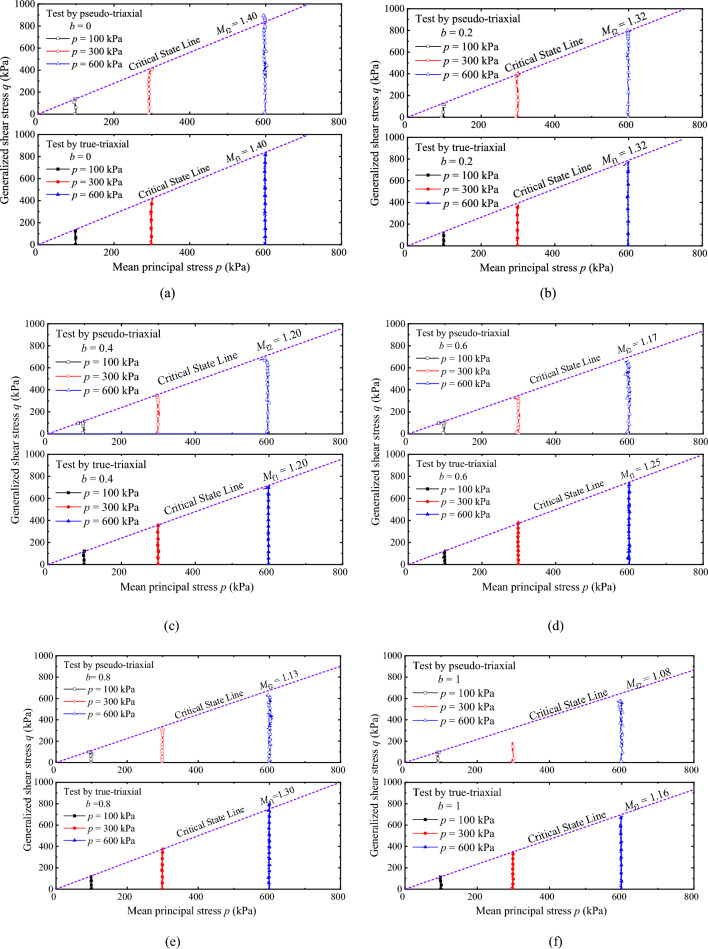
Comparison of effective stress paths under drained conditions: (**a**) *b* = 0; (**b**) *b* = 0.2; (**c**) *b* = 0.4; (**d**) *b* = 0.6; (**e**) *b* = 0.8; and (**f**) *b* = 1.

Figure 7a–f shows the effective stress path under drained conditions. The effective stress path measured by the PTA is calculated by the presented method, i.e., *p* = [(1 + *b*)*σ*_1_ + (2 − *b*)*σ*_3_]/3. The stress path measured in the TTA is the actual measured value. The critical state lines measured by the two apparatuses are drawn and the slope is marked in the figure. According to the analysis of Fig. 7a–f, the effective stress paths measured by the two apparatuses are loaded along the target value perpendicular to the *p*-axis, which can better complete the constant *p*-stress paths. Comparing the critical state points, it is found that the critical state points all pass through the critical state line (purple dotted line in the figure). Comparing the slope of the critical state line, it is found that the slope of the critical state line *M*_f2_ measured by the PTA is almost identical to the slope of the critical state line *M*_f1_ measured by the TTA when *b* = 0, 0.2, 0.4. At *b* = 0.6, 0.8, 1, *M*_f2_ is lower than *M*_f1_. Based on the *M*_f1_, the variation of *M*_f2_ at *b* = 0.6, 0.8 and 1 at the same *b* is calculated. At *b* = 0.6, the relative difference is 3.3%; at *b* = 0.8, the relative difference is 9.6%; at *b* = 1, the relative difference is 6.8%, and the variation range of the slope measured by the two apparatuses is less than 10%. The test results show that the critical state line measured by the two apparatuses has consistency when *b* is smaller, while when *b* is larger, the critical state measured by the PTA is lower.

**Figure 8:**
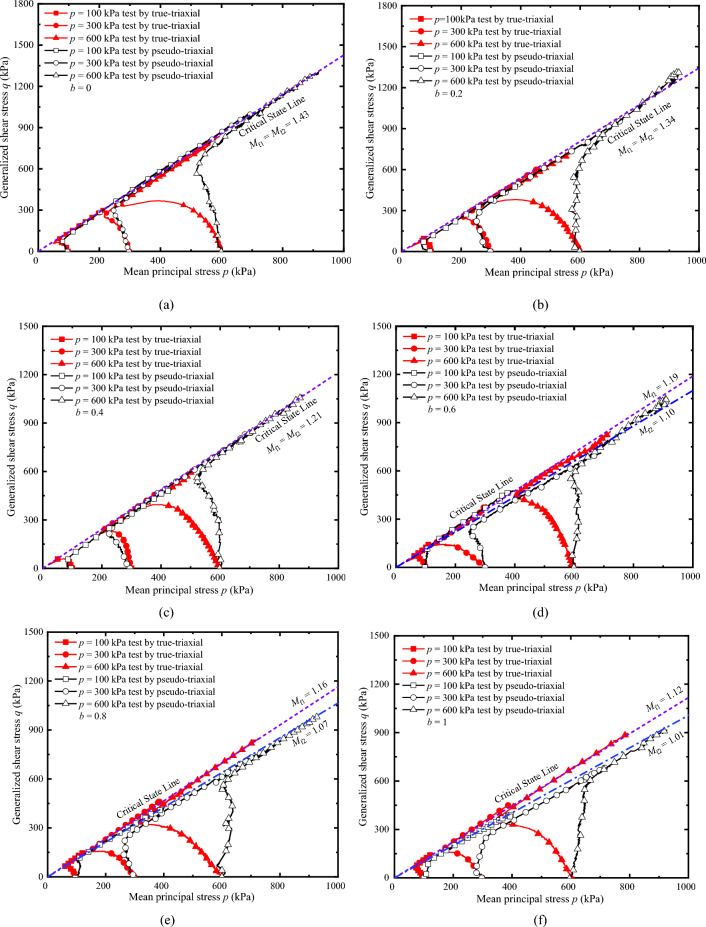
Comparison of effective stress paths under undrained conditions: (**a**) *b* = 0; (**b**) *b* = 0.2; (**c**) *b* = 0.4; (**d**) *b* = 0.6; (**e**) *b* = 0.8; and (**f**) *b* = 1.

Figure 8a–f shows the effective stress path under the undrained condition. The treatment method of effective stress path measured by PTA in the figure is consistent with that in Fig. 7. In fact, the effective stress path is drawn according to the effective stress principle, i.e. *p* = *p′* − *u*, where *p*, *p′* and *u* are effective mean principal stress, total mean principal stress and pore pressure respectively. The effective stress paths measured by both apparatuses are real sensor-recorded values, thus *u* = *p′* *− p*. Furthermore, the experiment was conducted under constant *p* conditions, and the evolution curve of *p* is equivalent to the pore pressure development curve. In addition, the critical state lines measured by the two apparatuses are drawn and the slope is marked in Fig. 8. Comparative analysis of Fig. 8a–f shows that the shape of the effective stress path curve measured by the two apparatuses under undrained conditions is significantly different, and the main reason for affecting the shape is the evolution of pore pressure, and the curve difference is more remarkable with the *b* increasing. This is due to the reduction of the confining pressure set by the PTA to ensure that the stress path is consistent with that of the TTA. Comparing the critical state points, it is found that the critical state points pass through the critical state line (purple dotted line), indicating that the specimens destroyed and critical state can be measured by the two different apparatuses respectively.

Comparing the slope of the critical state line at *b* = 0, 0.2, 0.4, the evolutions are completely consistent with the drained conditions. The variation range of *M*_f2_ with *M*_f1_ at *b* = 0.6, 0.8, 1 under the undrained condition compared with *M*_f1_ at the same *b* were calculated. At *b* = 0.6, the relative difference is 7.6%; at *b* = 0.8, the relative difference is 7.8%; and at *b* = 1, the relative difference is 9.7%, which is similar to the results measured by the drained test under the same conditions, and the variation range of the slope measured by the two apparatuses is less than 10%. The results of the effective stress path measured under drained and undrained conditions fully prove the feasibility and rationality of PTA to achieve the three-dimensional stress path. However, the PTA is not the actual TTA. It can only achieve part of the true-triaxial stress path in *p* − *q* space equivalently. There are still differences between the measured test results, which need to be corrected later.

### Comparison of stress–strain relationships

**Figure 9:**
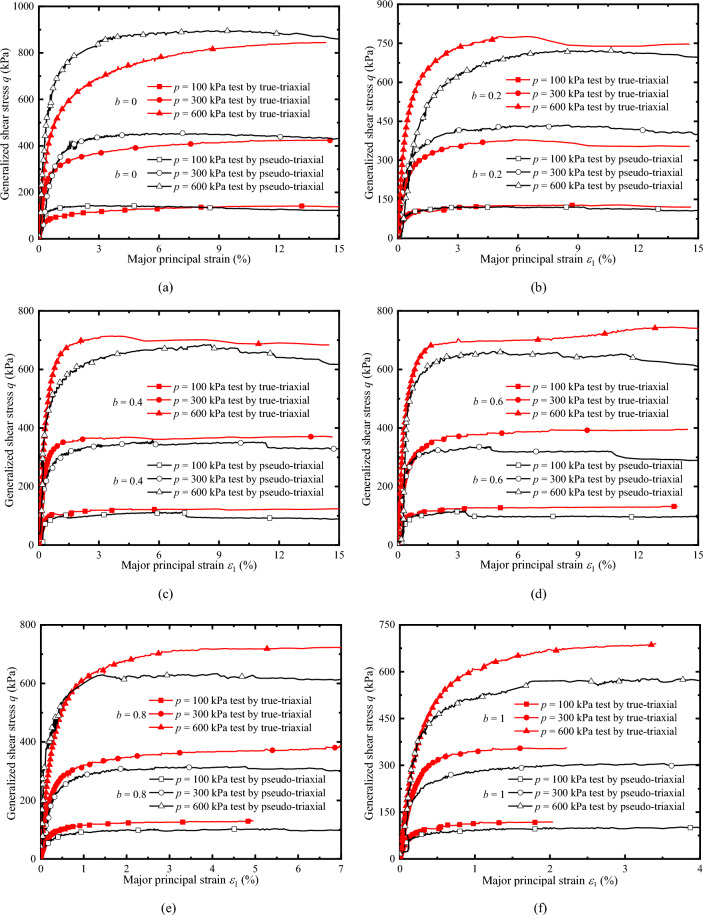
Comparison of stress–strain curves: (**a**) *b* = 0; (**b**) *b* = 0.2; (**c**) *b* = 0.4; (**d**) *b* = 0.6; (**e**) *b* = 0.8; and (**f**) *b* = 1.

Figure 9a–f compares the generalized shear stress *q* and major principal strain *ε*_1_ measured by two apparatuses under drained conditions. Under the same condition, the shape of the *q* − *ε*_1_ curves measured by the two apparatuses has better similarity. When *b* is lower (*b* = 0, 0.2, 0.4), the shape of the *q* − *ε*_1_ curves are similar at different *p*, and the peak stress is almost consistent. When *b* is larger (*b* = 0.6, 0.8, 1), the shape of the *q* − *ε*_1_ curves has little difference, but its peak value differs. Except *b* = 0, *q* − *ε*_1_ curves measured by TTA are slightly higher than the *q* − *ε*_1_ curves measured by PTA, and the larger *b*, the greater the difference. At *b* = 0, the measured curve is just the opposite. Although there are differences in the *q* − *ε*_1_ curves in Fig. 9, the curves have higher similarity in the whole, indicating that the triaxial test adopting the presented method can equivalently achieve part of the three-dimensional stress path, and the measured test results have a certain reference value.

**Figure 10:**
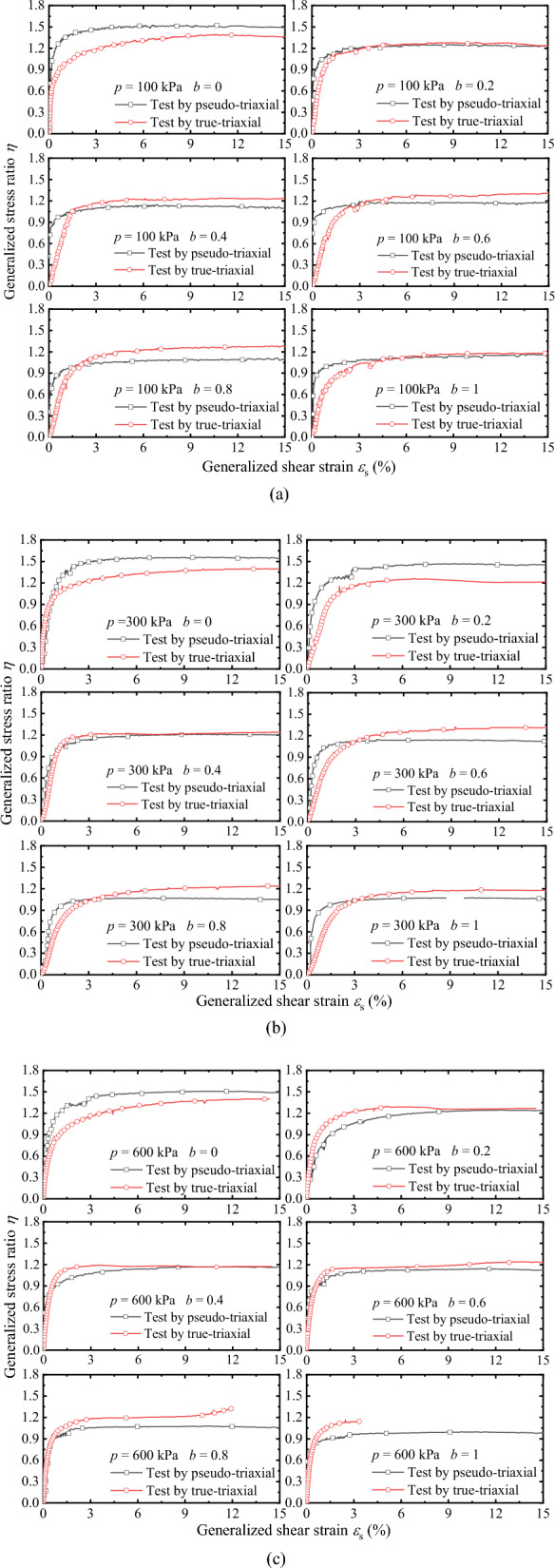
Comparisons between generalized shear strain and generalized stress ratio under drained conditions: (**a**) *p* = 100 kPa; (**b**) *p* = 300 kPa; and (**c**) *p* = 600 kPa.

Figure 10a–c are *η* − *ε*_s_ curves measured by two apparatuses under drained conditions, where *η* = *q*/*p*. It can be drawn from the comparison and analysis of the information in the figure that when *b* is lower (*b* = 0, 0.2, 0.4), the *η* − *ε*_s_ curve is similar, and the peak points of *η* are almost consistent. When *b* is larger (*b* = 0.6, 0.8, 1), the* η* − *ε*_s_ curves and peak points of *η* are different, i.e. the peak value of *η* measured by TTA is higher than that measured by PTA. Similar to the stress–strain curves in Fig. 9, except *b* = 0, the *η* − *ε*_s_ curves measured by TTA are slightly higher than that measured by PTA, and the larger *b*, the greater the difference. However, the *η* − *ε*_s_ curves measured by TTA are slightly lower than that measured by PTA at *b* = 0.

**Figure 11:**
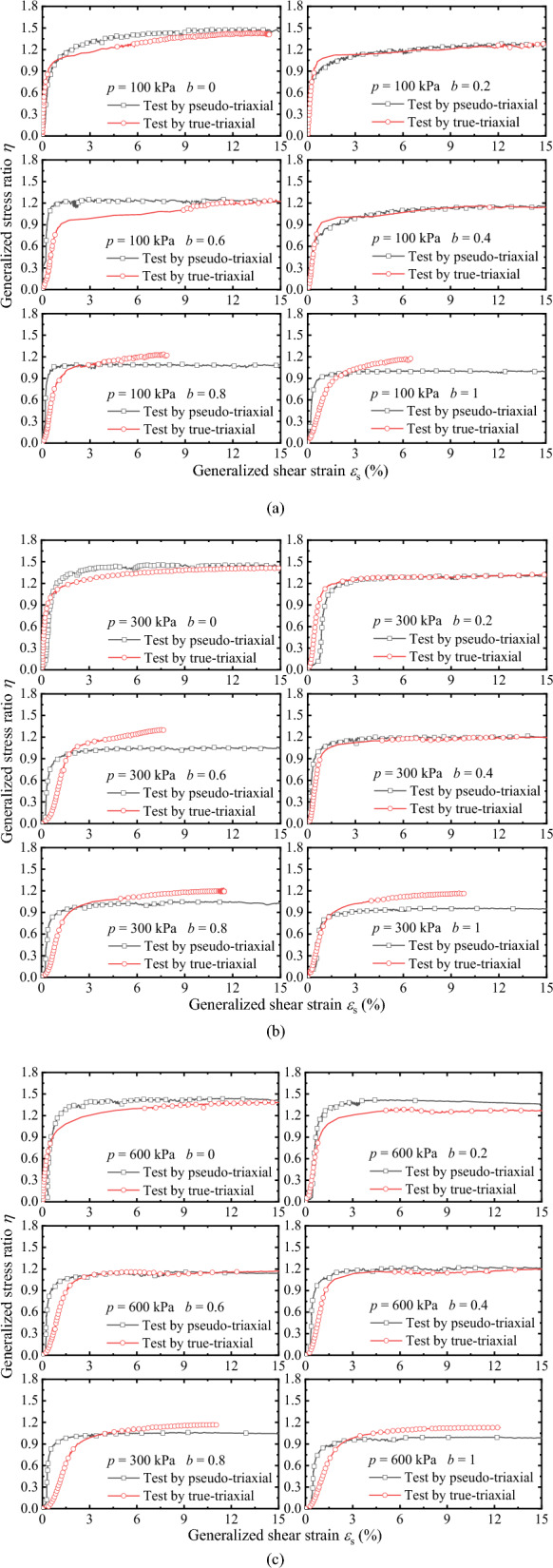
Comparison *η* − *ε*_s_ curves under undrained condition: (**a**) *p* = 100 kPa; (**b**) *p* = 300 kPa; and (**c**) *p* = 600 kPa.

Figure 11a–c shows the *η* − *ε*_s_ curves measured by the two apparatuses under undrained conditions. On the whole, the *η* − *ε*_s_ curves are consistent with the evolution under drained conditions. However, compared with the *η* − *ε*_s_ curves, the elastic modulus of the *η* − *ε*_s_ curves under the undrained condition is lower than that under the drained condition. When *b* is larger (*b* = 0.6, 0.8, 1), the *η* − *ε*_s_ curve shape measured by the two apparatuses is different, and the peak value of *η* is different. According to a qualitative analysis, the elastic modulus *E* (determined by the secant method, *E* = ∆*η*/∆*ε*_s_) of the *η* − *ε*_s_ curves measured by the TTA is lower than that measured by the PTA. Comparing the peak point of *η* under drained and undrained conditions, the peak value of *η* is slightly higher than in the drained condition. The *η* − *ε*_s_ curves prove that the presented method can be obtained stable and reliable data. The parameters can be applied in engineering and the establishment of strength criteria after correction.

### Comparison of strength on deviatoric plane

To reveal the peak strength characteristics of aeolian sand on the deviatoric plane, verify the feasibility of the presented method, and analyze the difference between the peak strength measured by the two apparatuses, we adopt the two popular criteria to predict the strength and compare the test peak points in this paper.

First, the general equation of the Mohr–Coulomb criterion is written as follows.

7 $$q - M_{f} g\left( {\theta_{\sigma } } \right)p = 0$$

where *M*_*f*_ is the peak stress ratio measured by PTA. *g*(*θ*_*σ*_) is a shape function. The expression of *M*_*f*_ is as follows.

8 $$\left\{ \begin{gathered} M_{f} = \frac{{6\sin \varphi_{f} }}{{3 - \sin \varphi_{f} }}\left( {b = 0,\;\theta_{\sigma } = - 30^\circ } \right) \hfill \\ M_{f} = \frac{{6\sin \varphi_{f} }}{{3 + \sin \varphi_{f} }}\left( {b = 1,\;\theta_{\sigma } = 30^\circ } \right) \hfill \\ \end{gathered} \right.$$

where *φ*_*f*_ is the peak internal friction angle.

The expression of the first shape function^30^ is as follows.

9 $$g\left( {\theta_{\sigma } } \right) = \frac{{2\left( {1 - c^{2} } \right)\cos \left( {\frac{\pi }{6} - \theta_{{_{\sigma } }} } \right) + (2c - 1)\sqrt {4\left( {1 - c^{2} } \right)\cos^{2} \left( {\frac{\pi }{6} - \theta_{\sigma } } \right) + c(5c - 4)} }}{{4\left( {1 - c^{2} } \right)\cos^{2} \left( {\frac{\pi }{6} - \theta_{{_{\sigma } }} } \right) + (2c - 1)^{2} }}$$

where

10 $$c = \frac{{\left( {M_{f} } \right)_{{\theta_{\sigma } = 30^\circ }} }}{{\left( {M_{f} } \right)_{{\theta_{\sigma } = - 30^\circ }} }}$$

The expression of the second shape function is as follows^31^.

11 $$g\left( {\theta_{\sigma } } \right) = \frac{\sqrt 3 c}{{\left( {c + 1} \right)\cos \theta_{\sigma } + \sqrt 3 \left( {c - 1} \right)\sin \theta_{\sigma } }}$$

**Figure 12:**
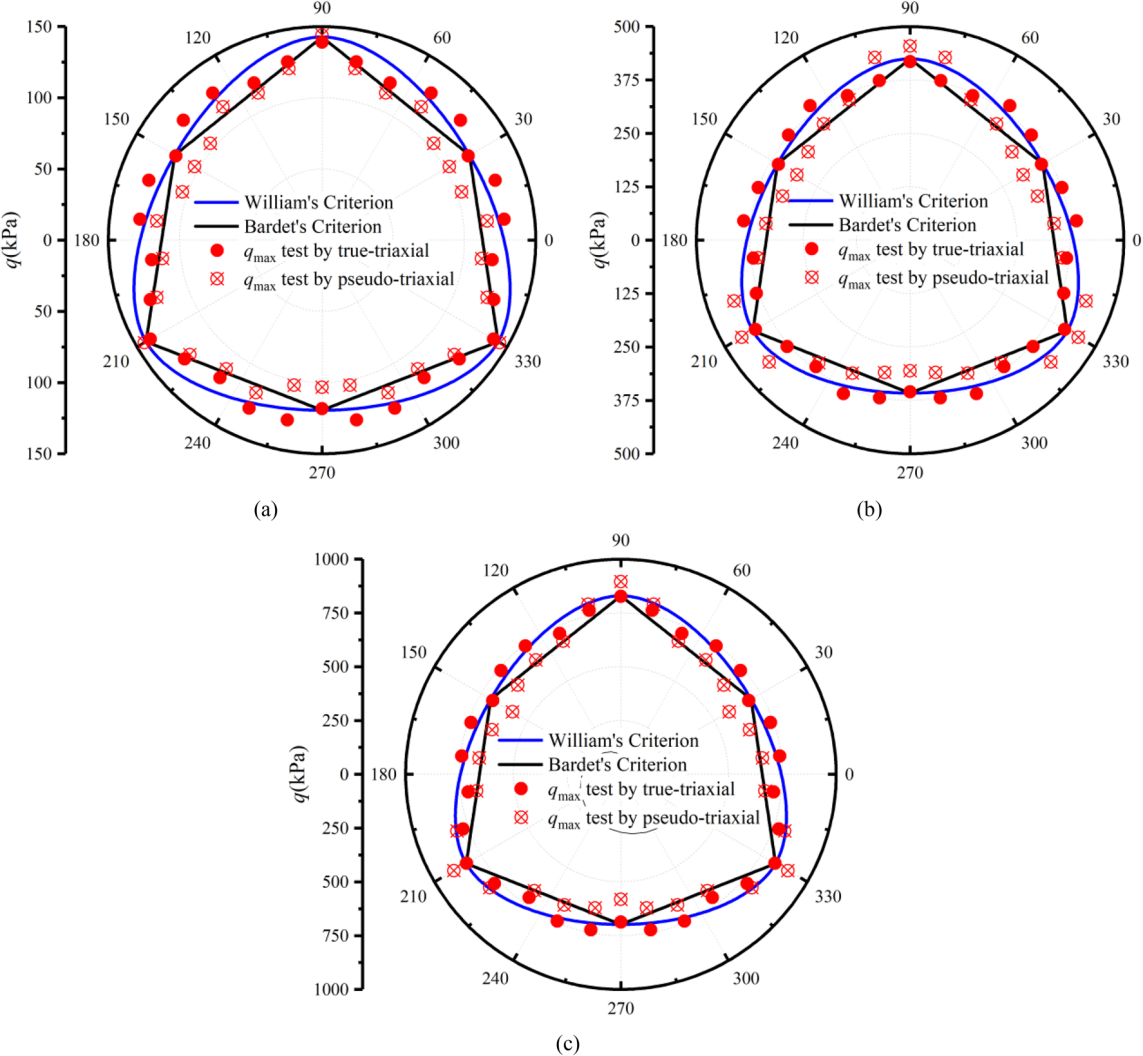
Comparison of peak stress points: (**a**) *p* = 100 kPa; (**b**) *p* = 300 kPa; and (**c**) *p* = 600 kPa.

**Figure 13:**
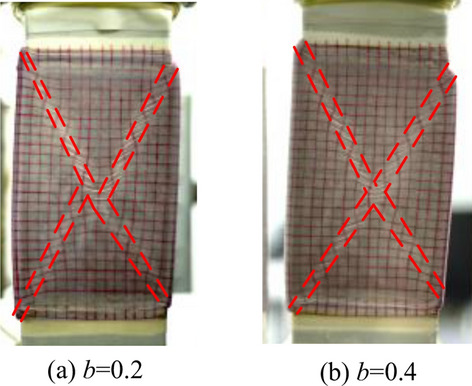
Shear band: (**a**) *b* = 0.2; and (**b**) *b* = 0.4.

Figure 12a–c shows the measured peak strength with TTA and PTA under drained conditions, and the predicted strength lines of the Mohr–Coulomb criterion modified by William and Bardet on the deviatoric plane based on the test parameters of TTA at *b* = 0 and *b* = 1. It can be drawn from the figure that the peak stress points measured by the two apparatuses under the same stress path are distributed near the Mohr–Coulomb criterion. Under the true-triaxial condition, the intermediate principal strain at *b* = 0.2 and *b* = 0.4 is smaller, which is close to the plane strain test condition. Under this condition, it is easy to produce strain localization, which leads to lower peak strength. During the test, we observed the deformation of the specimen and indeed the formation of shear bands, as shown in Fig. 13.

**Figure 14:**
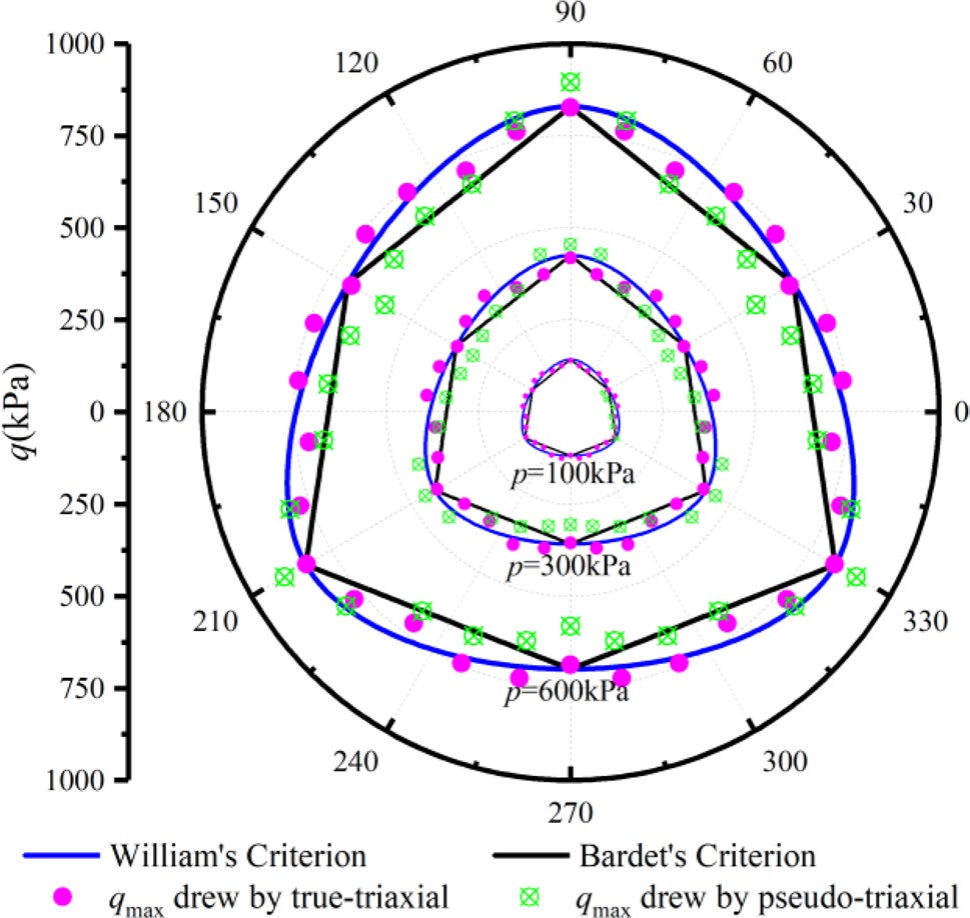
Comparison of average peak stress points.

On the whole, the peak stress measured by PTA is slightly lower than that measured by TTA. Only at *b* = 0 and *b* = 0.2, the peak strength measured by PTA is slightly higher than that measured by TTA. In the Fig. 14, the peak points measured by the two apparatuses are obtained by the multiplication of the average peak stress ratio *M*_*f*_ and average* p* under the three confining pressures, i.e., *q*_max_ = *M*_f_ × *p*. This point is the average value of the three groups of data, the average data is more stable, and the reference value is higher. The results better reveal the difference between the results of the two apparatuses and fully prove the feasibility and rationality of the presented method. This method breaks the traditional idea that the PTA can only study the stress path in two-dimensional space, in this paper, we have studied the stress path in three-dimensional space by PTA to achieve some functions of the TTA.

## Conclusions

To verify the feasibility and rationality of the presented method of achieving triaxial stress path equivalently by pseudo-triaxial apparatus, a series of drained and undrained true-triaxial tests with constant *p* were conducted by pseudo-triaxial apparatus and true-triaxial apparatus, and analyses the differences of results measured by the two apparatuses. The main conclusions are as follows.

1. The pseudo-triaxial apparatus can stably achieve the true-triaxial stress path with constant *p* under different *b* loaded by the presented method, and it is almost consistent with the stress path measured by the true-triaxial apparatus. The results vertify that the method is feasible and stable to control the loading path, and it can effectively achieve the purpose of measuring the three-dimensional mechanical properties.
2. The critical state line and its slope measured by the two apparatuses are almost identical when *b* is lower. When *b* is larger, the slope of the critical state line measured by the true-triaxial apparatus is slightly higher than that measured by the pseudo-triaxial apparatus, and the slope difference is less than 10%. The test results have certain references and practicability.
3. The relationships of *q*-*ε*_1_ measured by the two apparatuses have better similarity. Except for *b* = 0, the curves of *q*-*ε*_1_ measured by true-triaxial apparatus are slightly higher than that measured by pseudo-triaxial apparatus, and the larger *b* is, the greater the difference is. In addition, the peak value of *η* measured by the two apparatuses is almost the consistent when *b* is lower, and the peak value of *η* measured by the true-triaxial apparatus is slightly higher when *η* is larger. The results of *η*-*ε*_s_ prove that the presented method can obtain stable and reliable data equivalently, and the parameters can be used for engineering practice and the establishment of strength criteria.
4. The comparison between the peak points measured by the two apparatuses and the two popular Mohr–Coulomb criteria indicate that the peak points are distributed near the line of the criterion. Overall, the peak stress measured by the pseudo-triaxial test is slightly lower, and only slightly higher when *b* = 0 and *b* = 0.2.

The test results accurately reveal the difference between the measurement results of the two apparatuses and fully prove the feasibility and rationality of the presented method. This method equivalently achieves some functions of the true-triaxial apparatus. If it can be popularized, it will greatly expand the testing range of the pseudo-triaxial apparatus and greatly reduce costs and simplifies the process of true triaxial test.

## Data Availability

The data used to support the findings of this study are available from the corresponding author upon request.
